# Early-life exposures and long-term health: adverse gestational environments and the programming of offspring renal and vascular disease

**DOI:** 10.1152/ajprenal.00383.2023

**Published:** 2024-05-02

**Authors:** Zoé Oulerich, Amanda N. Sferruzzi-Perri

**Affiliations:** ^1^Department of Physiology, Development and Neuroscience, University of Cambridge, Cambridge, United Kingdom; ^2^Agro Paris Tech, Université Paris-Saclay, Paris, France

**Keywords:** fetus, kidney, pregnancy, programming

## Abstract

According to the Developmental Origins of Health and Disease hypothesis, exposure to certain environmental influences during early life may be a key determinant of fetal development and short- and long-term offspring health. Indeed, adverse conditions encountered during the fetal, perinatal, and early childhood stages can alter normal development and growth, as well as put the offspring at elevated risk of developing long-term health conditions in adulthood, including chronic kidney disease and cardiovascular diseases. Of relevance in understanding the mechanistic basis of these long-term health conditions are previous findings showing low glomerular number in human intrauterine growth restriction and low birth weight—indicators of a suboptimal intrauterine environment. In different animal models, the main suboptimal intrauterine conditions studied relate to maternal dietary manipulations, poor micronutrient intake, prenatal ethanol exposure, maternal diabetes, glucocorticoid and chemical exposure, hypoxia, and placental insufficiency. These studies have demonstrated changes in kidney structure, glomerular endowment, and expression of key genes and signaling pathways controlling endocrine, excretion, and filtration function of the offspring. This review aims to summarize those studies to uncover the effects and mechanisms by which adverse gestational environments impact offspring renal and vascular health in adulthood. This is important for identifying agents and interventions that can prevent and mitigate the long-term consequences of an adverse intrauterine environment on the subsequent generation.

**NEW & NOTEWORTHY** Human data and experimental animal data show that suboptimal environments during fetal development increase the risk of renal and vascular diseases in adult-life. This is related to permanent changes in kidney structure, function, and expression of genes and signaling pathways controlling filtration, excretion, and endocrine function. Uncovering the mechanisms by which offspring renal development and function is impacted is important for identifying ways to mitigate the development of diseases that strain health care services worldwide.

## INTRODUCTION

Chronic kidney disease (CKD) is a progressive disease that has become one of the leading noncommunicable causes of death and is estimated to affect at least 10% of the population worldwide, amounting to almost 850 million individuals (1, 2). This high incidence can be explained by the increasing prevalence of associated risk factors, such as obesity, diabetes mellitus, and hypertension. However, epidemiological, clinical, and experimental studies have led to the hypothesis that the risk of developing chronic diseases in adulthood is also by environmental factors during critical periods of development and growth in early life (3, 4). This concept, known as developmental programming, was first formulated by Barker and Osmond (5) after finding a strong correlation between deaths from coronary heart disease and the neonatal death rate ∼60 yr earlier. They went on to demonstrate a similar association between low birth weight and an elevated risk of the individual developing cardiovascular diseases later in life (6). The link between suboptimal intrauterine conditions [indicated by neonatal death, intrauterine growth restriction (IUGR) or low birth weight] and increased long-term health complications has subsequently been confirmed by numerous studies and led to the emergence of the Developmental Origins of Health and Disease (DOHaD) hypothesis. Research in this area is thus of capital importance to better understand the significance of pre- and perinatal environments for normal development and later life disease risks. However, due to ethical constraints and the relatively long human life span, the use of animal models is necessary to bridge this knowledge gap.

The aim of this review is to summarize current knowledge about the consequences of adverse gestational environments on long-term renal and vascular health in offspring by successively exploring the roles of maternal nutrition, ethanol intake, gestational diabetes, glucocorticoid exposure, toxins, and hypoxia.

## THE RENAL AND CARDIOVASCULAR SYSTEMS ARE CLOSELY INTERTWINED

**Figure 1.**
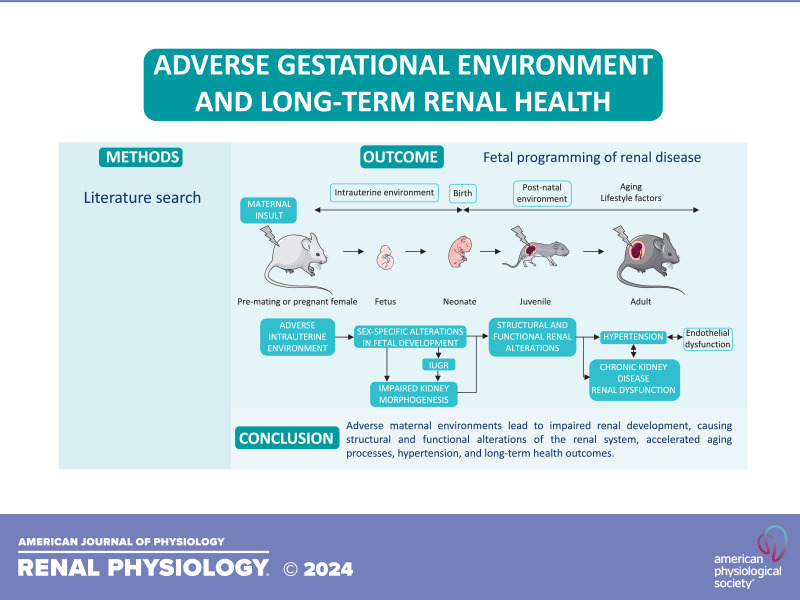
The renal and cardiovascular systems are closely intertwined. The renal system is responsible for blood filtration, fluid and electrolytes homeostasis, blood pressure regulation via the renin-angiotensin-aldosterone system, and plays a role in blood cells formation via the secretion of erythropoietin. This figure was partly generated using Servier Medical Art, provided by Servier, licensed under a Creative Commons Attribution 3.0 unported license (https://creativecommons.org/licenses/by/3.0/). ACE, angiotensin-converting enzyme.

When it comes to kidney development and function, much of our knowledge originated from studies of only two mammals, namely, rats and mice, in which development is comparable (7). Kidneys are highly vascularized organs that play a vital role in regulating blood volume and pressure, which in turn, are key determinants of cardiovascular function. In both humans and rodents, the kidneys receive around 20% of the cardiac output and maintain homeostasis by filtering waste products and retaining electrolytes, glucose, and proteins (8) (Fig. 1).

Concerning blood pressure, one of the key mechanisms involved in its regulation is the renin-angiotensin-aldosterone system (RAAS). This signaling pathway is upregulated when the kidney senses hypovolemia or hypotension. It results in the conversion of angiotensin I (ANG I) into angiotensin II (ANG II), a powerful vasoconstrictor, but also stimulates the release of aldosterone from the adrenal gland, which increases sodium reabsorption in the kidney; both mechanisms lead to an increase in both blood volume and pressure. The RAAS is tightly regulated, and disruption in this balance can lead to hypertension, heart failure, and kidney disease (9). Indeed, when inappropriately activated, the sodium retaining effects of ANG II necessitate increased blood pressure to maintain sodium balance via pressure natriuresis (10). During mammalian kidney development, the RAAS is crucial for various essential processes. First, its components, including renin and angiotensinogen, contribute to the mesenchymal to epithelial transition, a fundamental step in nephrogenesis. Second, ANG II promotes nephron formation by stimulating mesenchymal cell proliferation and inducing the expression of crucial growth factors. Third, ANG II regulates the differentiation and elongation of renal tubular structures within the developing kidney. Moreover, the RAAS is involved in the development and remodeling of renal blood vessels, ensuring proper vascularization of the kidney to support nephron growth and function. Finally, the RAAS helps establish and maintain fluid and electrolyte balance, critical for overall renal homeostasis. Dysregulation of the RAAS during embryogenesis can lead to developmental abnormalities and impairments in renal function later in life (11).

Nephrons are the functional units of the kidney responsible for blood filtration. They are in the renal cortex, namely the outer part of the kidney. Nephrogenesis occurs in the second half of gestation, starting around embryonic day (E)12.5 in mice [term is ∼E20 (12)]. In humans, nephrogenesis in completed around 36 wk of gestation, whereas in mice, most nephrons form after birth (12). Indeed, approximately half of all nephrons are formed between two and four days after birth. This means that in mice, full renal function is not established until a few weeks after birth. Final nephron number varies between mouse strains and is highly dependent on the number of cell progenitors (13). In rats (term is ∼E23), morphological maturation occurs postnatally as well, with distinct regions reaching maturity at different postnatal days (PND). The papilla matures earliest (PND7), followed by the cortex (PND21), and finally the medulla (PND30). Concerning nephrogenesis, new nephrons continue to form continuously until PND4, whereas previously established nephrons progress to complete their development up until PND15. As a result, all stages of nephron development co-exist within the kidney during the first postnatal week in rats (14). Thus, the quality of the pre- and perinatal environment is highly relevant for nephrogenesis, and reduced nephron number due to an environmental insult operating during these windows is strongly associated with a risk of increased blood pressure (15) and kidney disease (16, 17) in later life. In support of this, Brenner et al. (15) suggested that a congenital nephron deficit results in an impaired fluid and electrolyte balance and leads to volume expansion and adaptive glomerular hyperfiltration, which may, over time, leads to glomerular injury and hypertension. The relationship between low nephron number and hypertension and renal disease, also called the Brenner hypothesis, is however debated. This is because nephron number per kidney can vary more than 10-fold between healthy individuals, ranging from 200,000 to over 2.5 million (18). In addition, human studies are limited with a focus on investigations of individuals born with relatively low nephron numbers (19), and nephron number is not associated with adult blood pressure in some populations, e.g., African Americans (20). Furthermore, a sudden reduction in nephron numbers, for instance in kidney donors does not always cause worsened cardiovascular or renal outcomes (21).

## THE IMPACT OF A SUBOPTIMAL IN UTERO ENVIRONMENT ON CARDIO-RENAL HEALTH

### Maternal Undernutrition

#### Protein restriction.

Protein restriction during gestation is the most widely used experimental insult to study the effect of early nutrition on renal development, with the rat being the most studied animal model. Low-protein diets (LPDs) usually contain 8–9% protein in the form of casein, however, some studies reduce it down to 6%, with the control diet varying from 17% to 22% protein content (22, 23). Diets are usually made isocaloric by increasing the proportion of carbohydrates and lipids.

A maternal LPD has been shown to result in low birth weight in rats (23–29) with a greater reduction in birth weight observed with either a more restrictive LPD [a 30% reduction in birth weight was observed when dams were fed a 6% protein diet (23)] or when the LPD was fed for a longer period [24% to 39% reduction in birth weight when the LPD was fed from up to 3 wk before mating until lactation (24, 26)]. Interestingly, LPD in mice (30) or sheep (31) does not result in a reduction in fetal weight, and one study in rats found heavier offspring at birth from LPD-fed mothers (32). The explanation for species and study differences is unclear. Variability in the precise composition of the LPD and control diet used, and timing and duration of the dietary intervention between species and studies may explain some of the discrepancies between birthweight outcomes. There may also be a role for differences evoked in gestational tissues by the LPD. Previous work has shown there is upregulation of glucose transporters in the placenta (33) and yolk sac (30) when maternal protein intake is restricted in mice that may underlie the lack of a change in birthweight (30). While in rats, there is no adaptive upregulation of placental glucose transport and placental amino acid transport is reduced and fetal growth decreased by a LPD (34). Concerning sheep, the explanation could lie in a greater metabolic reserve compared with rodents, which allow them to be more able to metabolically adapt to nutritional deficit. Moreover, being uniparous, the metabolic demands required to support gestation in sheep are relatively lower than in the multiparous rodents (35).

Offspring of protein-restricted mothers remained lighter at 12 wk (23), 6 mo [by 15% (26)], and 32 wk [by 10% (24)] even though weight differences were no longer observed at 100 wk (24). These data may indicate a catch-up in growth of the LPD offspring later in life, or increased age-related fat deposition due to metabolic changes. Kidney weight was reduced in adulthood in most studies of LPD (23, 24, 26, 30), whereas kidney-to-body weight ratio was either decreased at birth (25) or in adulthood (24, 26–28), increased in adult life (23), or unchanged in both fetal and early life (31, 32). There were also some sex-specific differences reported in the offspring of LPD mothers (24, 27, 36). For instance, body and kidney weights of males from LPD-fed mothers and weaned onto a control diet were lower at 5 wk and 24 wk, respectively, compared with controls, whereas females underwent catch-up growth and showed no changes (27). Furthermore, females from LPD mice showed glomerular hypertrophy at PND30 compared with males (36) and arterial and renal dysfunction at 100 wk compared with both female controls and male control and LPD groups (24). Another study (29) showed that a postweaning high-salt diet (HSD) increased the weight of kidneys from LPD offspring, although kidney weights were reduced at birth when compared with control offspring. This latter study highlights the interaction between the pre- and postnatal environment, and how developmental programming effects may be exposed or amplified by a subsequent adverse postnatal condition.

Other studies have shown that a maternal LPD resulted in reduced nephron number in sheep fetuses at 65 days of gestation [term is ∼E145 (31)], neonatal (25), and adult rats (23, 26, 29, 32), as well as adult mice (27, 36). In addition, this can be associated with a decrease in glomerular size or volume (26, 31). Interestingly, one study in adult rats (26) found that the reduction in glomerular size seemed to affect cortical (outer) glomeruli more than juxtamedullary (inner) ones, with a 45% reduction in cortical glomeruli size (26). Since glomeruli grow and differentiate in a concentric manner and cortical glomeruli are the last ones to form, these data suggest that maternal protein restriction particularly affected later stages of nephrogenesis. The reduction in glomeruli size with a maternal LPD was associated with a deficit in podocyte endowment for cortical glomeruli (26). Podocytes are cells that line the Bowman’s capsule, which surrounds glomeruli. Podocytes are responsible for blood filtration (7), and a deficit in podocytes has been associated with decreased renal function and CKD (37). In adult offspring of LPD mothers, the deficit in podocyte numbers was associated with increased *Desmin* mRNA expression (23), an early marker of podocyte damage. Furthermore, renal and podocyte damage were more pronounced in offspring from LPD mothers who were subsequently fed with a high-protein diet (HPD, containing 30% protein) after weaning, with kidney hypertrophy at 12 wk and podocyte injuries associated with reduced glomerular filtration rate (GFR) and proteinuria (23–26, 28, 31). Similar effects were seen for offspring of LPD mothers who were subjected to pharmacologically induced diabetes postweaning (27). However, overall, males seemed to be more affected than female offspring (24).

As mentioned earlier, a nephron deficit may be associated with a greater risk of hypertension (22–25, 30, 38) and this can be worsened with a postweaning HPD (23) or HSD (29). Moreover, hypertension was linked with vascular dysfunction in adult LPD mice (30) and rats (24), but no change in heart rate was observed (22, 24, 28). Instead, the development of hypertension was related to reduced renal expression of *Renin* (25) and ANG II type I and II receptors [*AT1R* (19) and *AT2R* (32), respectively] in male pups from LPD pregnancies. Interestingly, *AT1R* expression was instead increased in female offspring of LPD mothers (19). In sheep, a maternal LPD resulted in an increased rate of apoptosis and reduced expression of *Vegf* and other angiogenic markers in the fetal kidneys (31). This study highlights the sensitivity of the vascular and renal systems to prenatal insults.

#### Caloric restriction.

The 2022 UNICEF report revealed that in 12 countries hardest hit by global food and nutrition crises, 6.9 million pregnant or breastfeeding women and adolescent girls had acute malnutrition. This, in turn, can lead not only to pregnancy and birth complications, but also permanent and negative consequences for child development and long-term health (39).

Maternal undernutrition has been studied in animal models of caloric restriction. In sheep, caloric restriction of 30% from 6 wk before until 7 days after conception (40) had no effect on fetal or kidney weight in offspring at 55 days of gestation. Although the lack of change may reflect the relatively short and very early period of gestation when caloric restriction was imposed, the findings are consistent with that from a meta-analysis on the impacts of maternal undernutrition in sheep (41). In rats, a 50% reduction in maternal calorie intake from mid-gestation was associated with low birth weight and a slight reduction in kidney weight and an increased heart-to-body weight ratio in adulthood (42). However, the same dietary restriction imposed throughout rat gestation did not affect kidney weight but resulted in hypertension and endothelial dysfunction in adult offspring. Furthermore, a study on the spontaneously hypertensive rat (43) showed that a 50% caloric restriction throughout gestation aggravated hypertension and endothelial dysfunction and led to a nephron deficit in adult offspring.

In humans, the Dutch Famine birth cohort study provided a unique way to investigate the effects of prenatal undernutrition and the later onset of disease (44). Several studies have shown that babies born from mothers exposed to an average daily intake of 1,000 calories had threefold higher risks of developing coronary heart disease (45) as well as increased albuminuria and reduced creatinine clearance (46), which can be signs of glomerular damage and may lead to a reduced renal function (47). The severity of the effects was dependent on the timing of the maternal calorie restriction during pregnancy. Indeed, the adverse programming effects on renal function (46) and cardiovascular disease (45) were observed specifically in those exposed to famine during mid or early gestation, respectively. This finding is not surprising, as organogenesis takes place during early gestation, and is followed by the rapid expansion of nephron number during mid-gestation. With respect to effects on renal function, results remained significant even after adjusting for other variables [including offspring body mass index (BMI), blood pressure, and diabetes], reinforcing an important role for maternal undernutrition as a programming agent for the offspring (46). A transgenerational effect was also observed; babies from both women and/or men who had been exposed to famine in utero, were on average, heavier at birth and had a greater BMI in adulthood (48) in association with epigenetic changes linked with the famine (49).

Taken together, protein or caloric restrictions during gestation seem to affect body and kidney weight, glomerular number and/or size, and alter the expression of genes related to renal function, damage, and vascularization. Moreover, changes were linked with accelerated renal and vascular dysfunction in adulthood in a sex-specific and species-specific manner.

### Maternal Overnutrition

In our modern societies, the increasing consumption of a high-fat (HFD) and sugar-rich diet, often referred to as a “Western” diet, has led to a growing percentage of maternal obesity, as well as related pregnancy complications. Indeed, maternal overnutrition and obesity can have long-term effects on offspring health, although little is still known about the effect on kidney development and function. Results of experimental studies investigating the effects of maternal overnutrition are challenging to interpret, since the type and composition of the diet can vary significantly across studies [e.g., hydrogenated coconut oil (50), animal lard (51, 52)], as well as the precise fat content [ranging from 20% to 58% (50, 51)]. Diets have also been supplemented with vitamins (51, 52) or not. The effect of postnatal overfeeding has also been investigated, as well either by litter reduction (53) or by feeding the offspring the same high-fat and high-sugar diet (HFHS) as the mother (54).

In mice or rats, although most studies have shown no effect of maternal overnutrition (due to HFHS diet) on fetal weight (50–52, 55) or adult weight (51, 52, 55, 56), others observed microsomia in male rats with a 25% reduction in birth weight and a slow body weight gain after weaning (57). Fetal overgrowth at *gestational day 21* and macrosomia at birth have also been observed in rats born from mothers fed a diet containing 28% animal saturated fat from before mating and throughout pregnancy. This could result from upregulated placental mechanistic target of rapamycin (mTOR) activity and nutrient transport (58). In humans, meta-analyses showed a strong association between unhealthy diets during pregnancy (characterized by high intakes of processed, high-fat, and high sugar-containing foods) and low birth weight and higher risks of preterm birth (59). However, rodent offspring that were fed a postweaning HFD (54, 56) or overfed due to litter reduction postnatally (53) were heavier in adulthood than both control offspring and offspring from overnourished mothers but weaned onto a control diet. Interestingly, there was no effect on adult offspring kidney weight (50, 51, 53), but nephron number was shown to be increased by a maternal HFHS alone (55) or with postnatal overfeeding (53). Furthermore, a perinatal and postweaning Western-style diet (containing an increased amount of fat and fructose) in rats exposed to a maternal HFHS diet resulted in glomerulosclerosis, podocyte injury, and tubulointerstitial fibrosis along with an increased expression of inflammatory markers at 17 wk of age (54). These observations suggest that a high-fat postnatal diet may exacerbate the effects of maternal overnutrition on renal pathology and may increase the risks of renal injury in adulthood.

Maternal overnutrition was also shown to program the adult offspring for increased proteinuria (50, 53), and particularly albuminuria (54, 56), which was related to an approximately twofold higher urinary albumin excretion when compared with offspring from control mothers at 17 wk (54). Proteinuria, and particularly microalbuminuria, are traditional early markers of CKD and a reduced renal filtration barrier that can arise from podocyte injury (60). Interestingly, offspring from overnourished mothers who were fed a HFHS had a ∼4.5-fold higher urinary albumin excretion than control offspring, indicating early-life programming can be exacerbated by poor postnatal nutrition. Despite this, GFR, which is an indicator of decreased renal function and plasma flow, was not affected (53–55). These findings suggest that the presence of albuminuria, glomerulosclerosis, and tubulointerstitial fibrosis resulting from a prenatal HFHS diet, and indicating possible renal damage, were independent of alterations in renal hemodynamics. The primary cause of renal injury is likely due to inflammation of the renal parenchyma in these offspring. However, changes in GFR and mean arterial pressure occur naturally due to the aging process, and the offspring may be particularly vulnerable to age-related alterations and the progression of renal damage.

Offspring from HFD fed mothers have been reported to show reduced renin activity, especially in adult males (51), but aldosterone levels were normal (51). Another study (50) showed that a maternal HFD increased renal expression of angiotensinogen (*Agt*) and angiotensin-converting enzyme (*Ace*) and AT1R protein abundance in the 16-wk-old offspring, suggesting an inappropriate activation of the RAAS and/or an impaired sensitivity to the RAAS. In addition, these changes were related to elevated blood pressure in the offspring (50, 53). These data are consistent with other work showing that inappropriate activation of the intrarenal RAAS is linked with the development of hypertension via the increase of pressure natriuresis (61, 62), and CKD (63).

Overall, maternal overnutrition seems to affect RAAS activity and renal and cardiac function in adult offspring, which in turn, can lead to hypertension in adulthood. These effects tend to be worsened by a HFHS diet after weaning.

### Micronutrient Restriction

Micronutrient undernutrition during critical periods of development and growth has become an important health issue in developing and developed countries, particularly among pregnant women and children who have an unbalanced diet. In experimental studies of offspring kidney development, data are available for maternal iron, sodium, and zinc restriction.

#### Iron restriction.

Iron is a vital micronutrient necessary for a diverse range of biological functions. During pregnancy, the mother needs to use her iron stores to supply the baby with the necessary amounts, which range from 500 to 800 mg for a normal singleton pregnancy (64). However, it is estimated that only 20% of women can meet this iron demand (65). Clinical data suggest that iron restriction during pregnancy can cause gestational anemia, reduce placental oxygen-delivery capacity, and can impair fetal growth and development, resulting in low birth weight, fetal distress, and preterm birth (66).

In rats, gestational iron deficiency [usually around 3 mg/kg compared with 52 mg/kg (67), 153 mg/kg (68), or 198 mg/kg (69)] has been linked with low birth weight in some studies (67, 69, 70) with either reduced (67) or unchanged kidney weight in adulthood (68, 70, 71). Interestingly, one study found increased kidney-to-body weight ratios after weaning (69). Moreover, iron restricted offspring were born small and remained lighter than control offspring (67, 68, 70, 71). In addition, one study reported that offspring from iron-deficient mothers born small underwent catch-up growth and were a normal weight by PND30–PND45 (69). Gestational iron deficiency was also shown to delay nephrogenesis in rats (69) and caused a nephron deficit in adults (68) independent of a change in kidney, cortex, or glomerular size (68). Sex-specific responses of renal morphology to iron restriction were observed, as adult males exhibited signs of glomerular basement membrane thickening and interstitial and glomerular collagen deposition and this was likely secondary to the increased blood pressure seen. Furthermore, programming effects were amplified by feeding the offspring a HSD postweaning (70).

A nephron deficit in the exposed offspring was correlated with hypertension (67, 68), increased inflammation and oxidative stress (67), and dysregulation of the RAAS with increased *Ang I* and *At1r* expression in the renal cortex and reduced *At2r* in adulthood (67). Moreover, it has been reported that prenatal iron deficiency increased both renal arterial pressure and sensitivity of arterial pressure to changes in dietary sodium intake, indicating long-term changes in the intrinsic pressure natriuresis mechanism (71). Surprisingly, one study found lower blood pressure in offspring from iron-restricted mothers (69). This discrepancy could be due to the age of the offspring when the measurements were conducted [6 wk (69), 3 and 16 mo (68), or 16 wk (67)]. Nonetheless, there are some data from humans that found lower blood pressure in 7-yr-old children born from mothers with anemia (72). However, iron levels and anemia were not assessed in these children, and so distinguishing between the effects of prenatal versus postnatal iron deficiency is not possible.

These studies together suggest there is a link between iron deficiency during pregnancy and impaired nephrogenesis and RAAS activity, which may increase the risk of the offspring developing hypertension in adulthood. Although data suggest male offspring may be particularly vulnerable to developing vascular dysfunction in response to both prenatal iron deficiency and HSD (73), further studies are required as research on maternal iron deficiency and offspring kidney and vascular health seems limited.

#### Zinc restriction.

Zinc plays a role in cellular growth, cell proliferation, and apoptosis, as well as in the activity of numerous zinc-binding proteins. Zinc deficiency is very frequent in low-income populations with low access to zinc-rich food such as meat, fish, nuts, and seeds. The importance of zinc nutrition in public health is now globally recognized, as zinc deficiency has been established as a leading factor of morbidity and mortality (74). Human studies have associated zinc deficiency in adults with a higher risk of developing hypertension (75), although clinical studies linking zinc deficiency during pregnancy and renal diseases in adults are difficult to find.

Concerning experimental studies, zinc restriction in pregnant dams resulted in low birth weight (76–78) and offspring remained lighter than controls postnatally. Both young and adult offspring also presented with reduced kidney weight, size (77, 78), nephron number, and GFR (76, 78). Sex-specific renal and vascular changes were also seen with prenatal zinc deficiency; male offspring showed reduced glomerular capillary area and increased renal apoptosis, hypertension, and hypertrophic remodeling of renal cortical arteries, whereas no changes were observed in females (76, 77). Moreover, RAAS genes showed sex-specific changes in expression before and after weaning (76, 77) with an increase in *Ang II* and *At1r* expression seen specifically in males. Males kept on a zinc-deficient diet after weaning showed more pronounced renal fibrosis, proteinuria, and oxidative stress damage than control offspring (76, 78). Furthermore, a control diet after weaning was unable to fully overcome the changes induced in the offspring by gestational zinc deficiency (78). Together, these data indicate that zinc deficiency during critical developmental windows during gestation can impair renal morphology, kidney function, and RAAS capability in offspring. These changes have the capacity to program the offspring for hypertension in the longer-term.

#### Sodium restriction.

Sodium restriction during gestation seems to have been less studied than sodium overload, and results seem to be as variable as the sodium content itself, ranging from 0.03% (79, 80) to 0.15% (81, 82) compared with a control intake of 0.5% (83) to 2% (82). In rats, these studies showed no effect on adult kidney weight with mild restriction (82), whereas birth weight was either unchanged (82) or reduced (81). Renal morphology and glomerular number were not affected by sodium restriction neither at 1 wk (81) nor at 12 wk (82). However, more severe restrictions in sodium intake [0.03% (79, 80) to 0.07% (83)] were associated with a 20% reduction in fetal weight with reduced fetal kidney weight in a few (79, 80), but not in all studies (83). Similarly, there are inconsistencies about whether sodium restriction during gestational development is linked to reduced nephron number (83) or not (79). Other work has shown that mild maternal sodium restriction during pregnancy induced a reduction in AT1R and AT2R abundance, decreased urinary sodium, and increased plasma urea in male pups, whereas reduced plasma aldosterone and sodium levels, increased creatinine, and reduced AT2R expression was seen in females (81). Maternal sodium restriction was also associated with a reduction in cardiac volume in males. Interestingly, this cardiac hypotrophy was linked with a complete absence of AT1R expression in the male, but not in female, neonatal heart (81). In other works, severe salt restriction resulted in elevated levels of fetal corticosterone and plasma renin activity (79, 82), as well as elevated blood pressure in adult offspring (79). Since sodium restriction during pregnancy has been associated with variable outcomes, it is difficult to conclude whether there is an effect of maternal sodium deficiency on offspring renal and vascular health. Further studies are thus needed to elucidate this gap in knowledge.

### Ethanol Intake

Alcohol is a known teratogen that can cross the placenta (84, 85) and thus may have direct and harmful impacts on the fetal growth, development, and survival. It is estimated that around 10% of women consume alcohol during pregnancy in any amount (84), and this prenatal alcohol exposure (PEE) can result in severe outcomes such as stillbirth (84, 86), premature birth, and low birth weight (87). The effect of alcohol depends on quantity/concentration, timing, and duration of the consumption during pregnancy (88).

Experimental studies have linked PEE to reduced fetal (89, 90) and postnatal weight (90) with unchanged (90, 91) or increased adult kidney-to-body weight ratio (89). When mothers were exposed for 21 h/day to a diet containing 15% ethanol-derived calories (90), fetuses in late gestation, especially females, had fewer nephrons with increased apoptosis levels compared with control offspring. Moreover, in male fetuses, PEE induced dysregulation of pro- and antiapoptotic factors and reduced the expression of the cell proliferation marker Ki-67 in the fetal kidney, as well as *Gdnf* and *Tgfb1* expression, which are important regulators of branching morphogenesis. These data hint at sexually dimorphic impaired renal morphogenesis in response to PEE. In rat PEE offspring, glomerular number and volume were decreased both at birth and in adulthood while creatinine levels were increased in adulthood (90). Even though renal artery function was not affected in PEE rat offspring (91), these data suggest that GFR was potentially reduced (89). Further work also revealed that PEE offspring exhibited an aggravated kidney damage response to a postweaning HFD, with severe glomerulosclerosis, tubular injuries, increased collagen deposition, and renal interstitial fibrosis observed (89). Protein expression of marker genes of podocytes, such as *Nephrin* and *Wt1,* was also decreased and PEE increased podocyte epithelial to mesenchymal transition, which is a marker of glomerular injury. There was also an increase in 11β-hydroxysteroid dehydrogenase 2 (11β-HSD2) and lower 11β-hydroxysteroid dehydrogenase 1 (11β-HSD1) in PEE offspring fed HFD, which would reduce glucocorticoid action on the kidney. Compared with the control offspring fed HFD postweaning, AT1R was increased and AT2R decreased in the HFD diet fed PEE male offspring, but reverse changes were seen in the female PEE group (89). Furthermore, glucocorticoid receptor (GR) was elevated, and insulin-like growth factor-1 (IGF1) and its receptor (IGF1R) were decreased in PEE males, with the opposite changes found in PEE females (89), which suggest a sex difference in the changes of the glucocorticoid-IGF1 axis in the adult offspring kidney in the PEE group. IGF1 is a downstream target of glucocorticoids and is involved in kidney metabolism and growth. Indeed, downregulation of the IGF1 signaling pathway has been reported to cause developmental retardation of the kidney (92). These data together highlight a sex-specific renal toxicity of PEE that involves multiple pathways.

### Gestational Diabetes

Like maternal obesity and overnutrition, maternal diabetes (type 1, type 2, and gestational diabetes) increases the risk of fetal growth problems and programs an elevated susceptibility of the child to develop metabolic syndrome (93), abnormal glucose tolerance, and type 2 diabetes (94) during childhood or adolescence. However, epidemiological studies linking maternal diabetes, impaired nephrogenesis, and renal diseases are lacking.

Concerning experimental studies, gestational diabetes can be induced using streptozotocin, a chemical with selective toxicity to pancreatic β-cells (95). The duration and gestational timepoint at which diabetes is induced in the mother (e.g., pregestational and gestational) overall seems to determine the severity and extent to which renal and vascular outcomes are affected in the offspring. When mild diabetes is induced before mating (96) or on the first day of pregnancy (97, 98), male rat offspring from mothers with diabetes showed a normal birth weight but exhibited greater weight gain over time (96, 98) in association with enhanced kidney growth (from 3 mo until 12 mo compared with control offspring) (96). However, when severe diabetes was induced in mice, offspring from mothers with diabetes were born small and remained lighter over time (97). Early glomerular hypertrophy was observed in these diabetes-exposed offspring, however, this was then associated with accelerated nephron loss throughout adulthood (96, 98), reduced GFR (96, 98), vascular dysfunction, and impaired tubular acid excretion (96). Exposing the growth-restricted offspring from mothers with severe diabetes to HFD (97) or HSD (98) postnatally resulted in rapid weight gain, catch-up growth, and worsened the effect of maternal diabetes on the kidney (97, 98). There was also enhanced activation of transforming growth factor-β1 (TGFβ1) and collagen type IV expression, increased oxidative stress, and enhanced lipid deposition in the offspring kidneys, suggestive of glomerulosclerosis. Albuminuria and proteinuria were increased and GFR was reduced in maternal diabetes exposed offspring. Offspring of mothers with diabetes were hypertensive (97, 98) and showed a greater sensitivity blood pressure dysregulation with a postnatal HSD (98).

When induced around mid-pregnancy in the mouse (E13), gestational diabetes also induced intrauterine growth restriction in offspring with a 20% reduction in fetal weight (99, 100). Kidney weight, glomerular size, and nephron number were also reduced in neonates (100), associated with nephron loss due to early apoptosis (100) and hypertension, which was more severe in adult male offspring (99). Extracellular matrix deposition and protein expression were increased in glomeruli (99). Moreover albuminuria (99), markers of oxidative stress (100) and inflammation were also seen in exposed male offspring (99). The expression of RAAS genes was also upregulated in the kidney of both neonates and adult offspring (99, 100). When mothers with diabetes were treated with insulin from E15 onward, low birth weight, hypertension, extracellular matrix accumulation, and RAAS genes dysregulation in the offspring were prevented (98).

Overall, animal studies report a nephron deficit associated with intrauterine growth restriction in offspring exposed to maternal diabetes, although these models are limited to severe maternal hyperglycemia, which does not reflect the typical clinical condition. Indeed, the streptozotocin-induced type 1 diabetes resulted in glycemia ranging from four times (98) to 17 times the normal glucose concentration (97, 99, 100) and led to fetal growth restriction, which is not a frequently reported outcome in human diabetic pregnancy. In a model of glucose intolerance that mimics a milder hyperglycemia associated with insulin resistance and the absence of intrauterine growth restriction (101), offspring of leptin receptor deficient mice (*Lepr^db/+^*), which spontaneously develop glucose intolerance during gestation, showed an early nephron deficit with glomerular hypertrophy at 6 mo, although renal function was normal. This suggests that when maternal glucose tolerance is impaired in a similar way as in human diabetic pregnancies, kidneys may adapt morphologically by increasing total glomerular volume to restore a normal filtration surface and thus maintain a normal renal function in adulthood. However, this seems to contradict the Brenner hypothesis.

### Glucocorticoid Exposure

Glucocorticoids (GCs) are a class of steroid hormones secreted by the adrenal gland that are released in response to environmental stress, such as cold, starvation, or psychological stress. Their action is mediated by the binding to the intracellular glucocorticoid receptor (GR), which acts as a transcription factor (102) and regulates various biological processes such as inflammation, glucose metabolism, and normal cardiovascular function (103). During development, the fetus is supposed to be protected from maternal GCs, which are inactivated by the placental enzyme 11β-hydroxysteroid dehydrogenase (11β-HSD2). However, studies have found that the expression levels of this enzyme can be reduced under chronic stress, which can allow maternal GCs to cross the placenta and thus potentially affect the fetus (104). In human pregnancies, women at risk of preterm delivery are usually given synthetic GCs, including β- and dexamethasone, which bypass placenta 11β-HSD2, to accelerate fetal pulmonary maturation and reduce neonatal mortality and respiratory distress syndrome (105). Moreover, it has been confirmed that all the components of the glucocorticoid activation system were expressed in rat kidney and involved in the regulation of renal development (106, 107).

The effects of GCs in animal models are usually investigated using the synthetic corticosteroid dexamethasone (108), which is usually administered during late pregnancy (109–116). Interestingly, dams treated with dexamethasone have been shown to reduce their food intake and lose (or fail to gain) a normal amount of weight during pregnancy (113). This might explain why offspring are born lighter (108, 109, 113–115, 117) with nephron deficit (110, 112, 114, 117). However, this deficit was observed to be induced by GC exposure during specific developmental time-windows (110); adult male offspring of dams treated on *days 13* and *14* of gestation had no nephron deficit, whereas those of dams treated on *day 15* or after had up to 20% less nephrons than control offspring. These findings are consistent with other studies (111, 114) and suggest specific windows in renal development, from E15 that are particularly sensitive to the effects of GCs. One study found that kidney weight was reduced by 30% in pups born from dams treated throughout pregnancy (117), whereas studies on late-pregnancy GC administration did not see any difference in kidney weight (113–116). Dexamethasone treatment was associated with renal injury, glomerulosclerosis (110, 117), reduced GFR (113, 117), and increased albuminuria in the adult offspring (117). It was also associated with increased levels of oxidative stress (116), and *Tgfß1* (112) and apoptosis-related gene expression (116). Moreover, the expression of RAAS-related genes was dysregulated in sheep fetuses, along with an observed altered response of the offspring kidneys to ANG II in a model exposed to dexamethasone during prenatal development (111). Similarly, there are reports of increased renin, ACE, and angiotensinogen expression in the kidneys of rats exposed to prenatal GC (115). Other work has shown that prenatal dexamethasone exposure resulted in hypertensive adult offspring (108–115, 117), with only males affected in several studies (112, 114). Other studies have also shown a greater increase in blood pressure when gestational dexamethasone exposed offspring were fed a Western style diet after weaning (115). Impaired autophagy mechanisms could be linked with the increased oxidative stress found in the dexamethasone offspring fed HFHS, as autophagy dysfunction has been associated with defects in mitochondria, which are the main cellular source of oxidative stress (116). Thus, GC exposure during late gestation seems to affect nephrogenesis and results in kidney injury and hypertension later in life.

### Chemicals

Rapid industrialization has led to an inevitable increase in the exposure of populations to multiple chemicals and pollutants, some of which may affect fetal development and growth, with resultant programming effects on the renal and vascular systems.

#### Bisphenol A.

One of the most common environmental pollutants is the endocrine-disrupting chemical bisphenol A (BPA). BPA is a chemical found in some plastics that mimics the action of estrogens in the body. In the rat, maternal BPA exposure combined with HFD is known to increase oxidative stress levels in the kidneys of adult male offspring; an effect that was prevented by a prenatal resveratrol, an antioxidant treatment (118). Maternal BPA exposure plus HFD was also associated with hypertension in adults, linked to decreased nitric oxide (NO) availability and activation of the aryl hydrocarbon receptor (118). In the mouse, maternal BPA administration induced glomerular abnormalities, as well as impaired nephrogenesis (119). Interestingly, maternal BPA abolished the sex-associated differences in glomerular morphology seen in control offspring. For instance, cuboidal parietal epithelial cells of the Bowman capsule that are characteristically seen in male were found in female offspring (instead of squamous cells) (119). This may suggest, an endocrine disrupting effect of BPA such that sex-specific cell types in the kidney are altered. These data together hint at a harmful effect of BPA on kidney development, although further work is needed.

#### Nicotine.

Epidemiological studies have linked maternal smoking during pregnancy with a higher incidence of congenital urogenital malformations (120) and reduced kidney weight in third trimester fetuses (121). Experimental studies investigating the effect of nicotine, the main component of cigarettes, on fetal development found unchanged (114, 122) or reduced birth weight, although offspring weights normalized by adulthood (123). Although kidney morphology was not affected by prenatal nicotine exposure (114, 122), in the spontaneously hypertensive rat, nicotine exposure upregulated renal *Igf1* and *Igfr* expression (122), suggesting a programmed de-regulation of growth controlling pathways in the offspring. Compared with control spontaneously hypertensive offspring, those exposed to intrauterine nicotine showed a greater increase in blood pressure and reduced heart weight at 9 wk of age, which indicates a worsening effect of nicotine on hypertension (122). These data suggest that programmed alterations in the cardiovascular system induced by intrauterine nicotine exposure depend on the genetic background. However, these studies do not reflect the reality of the impacts of maternal smoking of offspring programming, since cigarettes contain over 4,000 different chemicals (124), including a high level of toxic substance that promote oxidative stress. Indeed, exposing mice to cigarette smoke twice daily from 6 wk before mating through to lactation was associated with albuminuria, oxidative stress, DNA damage, and reduced mitochondrial oxidative phosphorylation efficiency in the kidneys of resultant offspring (123). Thus, although nicotine alone can impair growth controlling pathways and blood pressure in hypertensive models, its effects in other models are difficult to describe and do not mirror the effects of smoking. Studies looking at the intrauterine exposure to chemicals taken in through vaping/e-cigarettes are warranted.

#### Heavy metals.

Heavy metals such as lead (Pb), cadmium (Cd), or mercury (Hg) are common environmental pollutants that can be detected in blood, serum, and urine. Epidemiological studies have shown that these metals tend to accumulate in the kidneys, mainly in proximal tubule cells, causing structural and functional damage (125, 126). Indeed, in chronic intoxication, inert, protein-bound heavy metals tend to conjugate with metallothionein and glutathione, which are then released into the blood by the kidney (and liver). These compounds are subsequently reabsorbed through endocytosis and activate oxidative stress and apoptosis pathways, leading to chronic inflammation, fibrosis, and renal failure (126). In rats, chronic exposure to heavy metals during pregnancy reduced birth weight of the offspring in a dose-dependent manner (127). It also increased kidney-to-body weight ratio and resulted in congestion of the renal medulla and upregulation of *Kim1*, which has a pathophysiological role in regulating tubular damage and repair (127). Heavy metal accumulation in the kidneys thus can cause renal defects in the offspring in a dose-dependent way. However, much further work is needed.

#### Other.

One study (114) tested different chemicals on offspring outcomes in rats. Namely, they studied perfluorooctane sulfonate (PFOS) and perfluorononanoic acid (PFNA), which are commercial chemicals with wide applications, atrazine, an herbicide, and arsenic, which is used for various industries. This study found there were chemical- and sex-specific effects on birth weight, with females being born lighter except following prenatal arsenic treatment. However, body weights of exposed offspring were similar by weaning when compared with control offspring (114). Kidney weight was not affected, although nephron number was reduced (114). Blood pressure was higher in PFOS, atrazine, and PFNA gestationally exposed male offspring by 7–10 wk, whereas female offspring showed elevated blood pressure at 10 wk for PFNA and arsenic, and at 37 wk for PFOS and atrazine. Prenatal PFOS and atrazine exposure were associated with elevated renal *Gr* gene expression, suggesting programmed changes in GC regulation.

Other chemicals, such as 2,3,7,8-tetrachlorodibenzo-*p*-dioxin (128) or melamine (129), have been reported to induce renal injuries. Maternal exposition to the former caused fibrosis associated with hydronephrosis lesions, increased diuresis, and left ventricular hypertrophy in the offspring kidney (128). The latter caused tubule dilatation and interstitial edema with an accelerated growth of the developing fetal kidney, as shown by the shifted proportion of primitive versus transitional/mature glomeruli compared with control offspring (129). Minocycline, an antibiotic, was also found to reduce GFR and increase blood pressure in exposed offspring (130), whereas maternal exposure to perfluorobutane sulfonate was associated with dysregulation of RAAS gene expression in the offspring kidneys (131). Overall, these data highlight a renal toxicity of these various chemicals, although the dosages and models used might not reflect the effects on human renal development.

### Placental Insufficiency

Fetal growth changes, including intrauterine growth restriction can result from utero-placental insufficiency, a condition where the amount of nutrients and oxygen that reach the developing fetus is reduced. In animal models, placental insufficiency can be induced by bilateral uterine artery ligation in late pregnancy. Using this method, placental insufficiency in pregnant rats (132–138), sheep (139, 140), and rabbits (141) resulted in reduced birth weight (132–138) and persistent growth restriction through to adulthood in rats (133, 136). Kidney weight was reduced in all models (132, 133, 135, 139–141), and in rat offspring fed a postweaning HSD, kidney weight was increased in both control and placental insufficiency groups (136). In sheep, studies using twinning versus umbilico-placental embolization to study placental insufficiency and intrauterine growth restriction (140) showed that kidney weight was more severely reduced in twinning than in umbilico-placental embolization (36% and 27%, respectively) and kidney-to-body weight ratio was reduced by 20% specifically in twins. Nephrogenesis was impaired by bilateral uterine artery ligation in rats and rabbits; findings showed reduced nephron number in both models (132, 133, 138, 141) associated with glomerular hypertrophy (132, 133, 137) and hypertension in rats (132, 133, 135, 136, 138), and a greater increase in blood pressure particularly in rat offspring fed a postweaning HSD (136). One study in sheep showed an increased fetal blood pressure after umbilico-placental embolization, but this did not persist to adulthood (139). In the rat, kidney function was only marginally affected by uterine artery ligation, with increased proteinuria found in two studies (133, 136) in accompaniment with increased circulating potassium and urea (133). These effects were amplified by postweaning HSD (136). Glomerulosclerosis (134) and renal fibrosis (133, 136) were also observed in rats exposed to placental insufficiency.

At the molecular level, one study in the rat found that placental insufficiency-associated fetal growth restriction was related to age and sex-specific alterations in offspring kidney *Vegf/*VEGF expression (137); expression was reduced at birth and at PND21 in both sexes, but increased at PND120 in female and not in male offspring whose *Vegf* expression was similar to PND120 controls. Another study (138) found placental insufficiency-induced fetal growth restriction was linked to the downregulation of mRNA and protein levels of cyclooxygenase-2 (COX-2), a key protein involved in nephrogenesis, cortical architecture, and prostaglandin production, and 11β-HSD2 in the offspring kidneys at birth. At PND21, mRNA and protein levels of 11β-HSD2, GR, and mineralocorticoid receptor (MR), which mediates aldosterone actions on salt and water balance, were downregulated, but COX-2 expression was no longer different compared with controls. These data together suggest that feto-placental insufficiency during late gestation impairs nephrogenesis and predisposes toward renal malfunction by affecting multiple pathways.

### Oxygen Supply

More than 140 million people live at altitudes over 2,500 m, which is the conventional definition of high altitude where arterial dioxygen (O_2_) saturation starts to decline. At this altitude, low oxygen availability can induce fetal hypoxia, which has been correlated to reduced fetal growth and birth weight (142, 143). However, maternal intermittent hypoxia can be experienced at lower altitudes due to maternal anemia, pulmonary disease, or smoking, which put the fetus’ health at risk.

Animal models of fetal hypoxia usually investigate the effect of either moderate [10–13% (144–148)] or severe [less than 7.5% (148, 149)] maternal inhalation hypoxia. When moderate maternal hypoxia was imposed throughout pregnancy (144, 146), fetal and kidney weights were reduced (146). Moreover, the offspring kidneys displayed enlarged Bowman and interstitial spaces with apparition of autophagic structures linked with an upregulation of autophagy-related genes (146). There are also reports of cardiac and peripheral vascular dysfunction in offspring exposed to hypoxia during gestation (144).

In the mouse, when transient, moderate hypoxia is imposed in mid-gestation (12% for 48 h from E12.5), fetal body and kidney weights were reduced as early as E14.5 and associated with congenital anomalies of the kidney and cells with wrinkled nuclear membranes [characteristic of hypoxic stress (150)], reduced nephrogenesis and uretic branching, downregulation of oxygen-responsive genes, and dysregulation of β-catenin signaling, indicating impaired renal morphogenesis (148). Moreover, studies investigating the effect of moderate hypoxia during the second half of gestation in mice (147) or late gestation in rats (145) showed reduced birth weight and reduced kidney weight, although these changes were no longer noticeable in 8-wk-old mice (147) and 3-mo-old rats (145). Nephron number was also reduced at birth, with a 40% reduction observable in E18.5 fetuses linked with nephron degeneration (necrosis and apoptosis) (147), reduced DNA replication and ribosomal activity, and increased mRNA degradation in the kidney. This reduction in nephron number progressively worsened in adulthood in a sex-specific way; females were more affected than males (52% vs. 26% reduction) (145). Furthermore, renal function was severely reduced at 15 mo, expression of fibrotic and inflammation markers enhanced (147), and ANG II and *At1r/*AT1R downregulated, with once again, a greater effect seen in female offspring (145).

In mice, severe acute hypoxia in early gestation (5.5 to 7.5% for 8 h from E9.5) resulted in reduced body weight and kidney length, and impaired renal morphogenesis in the offspring (147). Furthermore, in offspring exposed to prenatal hypoxia, impaired branching, unilateral or bilateral duplex kidneys, and dysregulation of β-catenin signaling were observed with partial penetrance (148). When mouse and rat embryonic kidney cultures were exposed to severe hypoxia [1–5% for 3–7 days (149) or 1–3% for 6 days (151)], nephron differentiation, tubule formation, and vascularization were impaired, which was consistent with observations seen in vivo. Thus, prenatal hypoxia can lead to structural and functional cardiovascular and renal defects with dysregulation of molecular pathways.

## CONCLUSIONS

**Figure 2.**
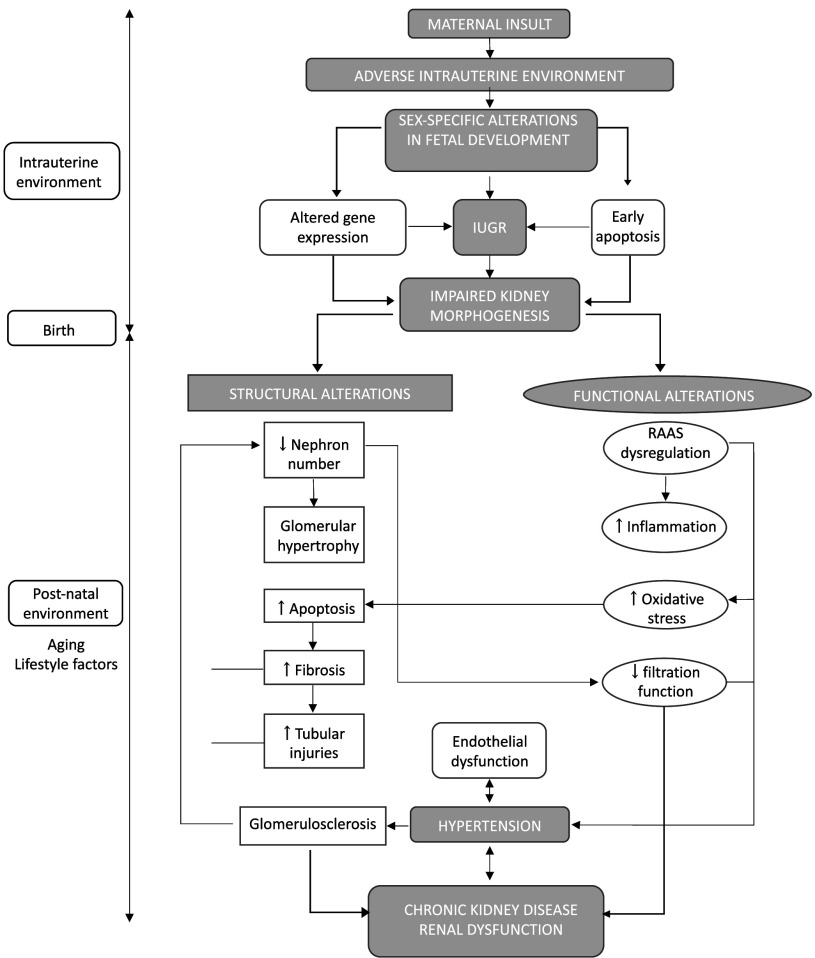
Fetal programming of renal disease. An adverse maternal environment leads to impaired renal development, causing structural and functional alterations of the renal system, accelerated aging processes, hypertension, and long-term health outcomes. IUGR, intrauterine growth restriction; RAAS, renin-angiotensin-aldosterone system.

Adverse intrauterine conditions can have long-term effects on cardiovascular and renal development. These changes may increase the risk of developing cardiovascular diseases, hypertension, CKD, and other related conditions in later life (Fig. 2). However, conclusions are not always easy to draw from experimental studies, as inconsistencies exist. The first source of inconsistencies comes from the difference in experimental animal used. Indeed, nephrogenesis varies in time and duration between species, and the same insult may not have the same outcome or severity in rodents and sheep, which do not have the same metabolism nor the same lifespan. Moreover, when studying long-term effects of adverse gestational environments, one must pay attention to time- and dose-dependent effects of certain insults, as chronic outcomes can arise from nonspecific mechanisms and secondary to lifelong metabolic perturbations rather than aberrant kidney development. Indeed, assessing early outcomes would provide a more definitive mechanistic link with the stressor and should be a key objective of future work. Many studies also observed effects at molecular and cellular levels in the kidney following adverse intrauterine conditions, although it is not always clear how these changes relate to renal function. Overall, renal outcomes require more time-course studies with multiple levels of observation in future studies.

Although sex-specific differences in renal physiology, growth patterns, and metabolic function can be observed, they are frequently overlooked, with either a predominant focus on males or an indiscriminate study of both sexes. However, epidemiological evidence indicates a notable discrepancy in the incidence and prevalence of renal diseases between males and females (152). Thus, both sexes should be studied separately in future investigations to determine whether the developmental programming of renal disease correlates with sex-specific gene expression patterns or variations in hormonal profiles. For example, experimental models could explore the impact of acute or chronic administration of sexual steroids on male and female neonates to link these alterations to the risk of developing renal pathology later in life.

Interestingly, some models of fetal programming can involve catch-up growth patterns. In the DOHaD field, the concept of catch-up growth is significant due to its implications for long-term health. Catch-up growth occurs as a compensatory mechanism after a period of stunted growth, such as intrauterine growth restriction following a maternal insult. The molecular and cellular mechanisms are complex and involve systemic and local mechanisms, including hormones, genetic and growth factors, environment, and nutrition (153). However, if offspring born with restricted intrauterine growth undergo excessive catch-up growth during the postnatal period, they can be predisposed to higher renal vulnerability due to the insufficient renal reserve capacity to accommodate the increased functional load. Moreover, evidence shows that catch-up growth increases the risks of developing metabolic abnormalities notably due to a dysregulation of genes related to metabolic and immune processes (154). Further work is required to assess if efforts to control or avoid catch-up growth in offspring (such as by restricting food intake) could mitigate long-term programming of renal and vascular disease.

Consistent with the catch-up growth phenomenon, emerging evidence indicates that the susceptibility to chronic diseases in adulthood is not solely due to adverse prenatal conditions, but also influenced by postnatal environmental exposures. As seen in this review, this is exemplified in the phenomenon of “second hits,” where postnatal stressors such as excessive salt intake/HSD or high-fat diets/HFD exacerbate both the metabolic and renal consequences of prenatal insults, including maternal caloric restriction or overfeeding. Prenatal insults can induce modifications in renal structure, nephron endowment, and genetic pathways in affected offspring. When coupled with persistent adverse environmental factors throughout life, these alterations are likely to exacerbate renal hemodynamic abnormalities and activate proinflammatory or oxidative pathways, thus accelerating renal aging and precipitating renal injury. However, only a few of the perinatal stressors considered in this review assessed the impact of a “second hit” like a HSD or HFD. Hence, further work is needed in this area and could also involve the combination of another stressor postnatally. Investigating the link between pre- and postnatal environments and their repercussions on long-term health outcomes is therefore imperative to better understand their influence on renal health and disease, and may give valuable insights for generating new therapeutic interventions.

Investigating the molecular and cellular mechanisms underpinning the programmed changes resulting from adverse gestational environments may help developing new possible treatments to improve cardio-vascular and renal outcomes. Thus, some studies have investigated different therapeutic interventions and their possible reversal effects on renal health following prenatal stresses. For instance, as hypertension, vascular dysfunction, and renal damage can be associated with oxidative stress (38), prenatal administration of antioxidants could prevent the programming effects of maternal undernutrition (38) or GC administration (116) on fetal susceptibility to oxidative stress and RAAS upregulation and improve vascular parameters later in life. Growth hormone has been shown to exert direct and beneficial effects on blood vascular structure and function (155), and studies have shown that growth hormone treatment reversed the effects of growth failure and the excessive mortality rate of children suffering from early CKD (156). Moreover, experimental studies on rats observed that preweaning growth hormone treatment reversed hypertension and endothelial dysfunction in adult male offspring of mothers undernourished during pregnancy (157). This could be mediated by the restoration of the circulating levels of IGF1, which are reduced at birth in the offspring of undernourished mothers (158) and have been shown to improve endothelial dysfunction in rats (159). However, much further work is required to explore the utility of different treatments, including the precise timing of administration and if treatments could be selectively targeted to the kidney or vasculature to maximize the benefit.

To summarize, further studies are needed to uncover the effects and mechanisms by which adverse gestational environments impact offspring renal and vascular health in adulthood. In doing so, we may be able to identify agents and interventions that halter and ameliorate life-long consequences of an adverse intrauterine environment on the subsequent generation.

## GRANTS

This work was supported by funding awarded to A.N.S.-P. from the Medical Research Council under Grant MR/R022690/1/RG93186 and the Lister Institute of Preventative Medicine under Grant RG39692.

## DISCLOSURES

No conflicts of interest, financial or otherwise, are declared by the authors.

## AUTHOR CONTRIBUTIONS

Z.O. and A.N.S.-P. conceived and designed research; Z.O. performed literature review; Z.O. analyzed data; Z.O. and A.N.S.-P. interpreted results of literature review; Z.O. prepared figures; Z.O. and A.N.S.-P. edited and revised manuscript; Z.O. and A.N.S.-P. approved final version of manuscript.
